# Dietary Flavonoids, *CYP1A1* Genetic Variants, and the Risk of Colorectal Cancer in a Korean population

**DOI:** 10.1038/s41598-017-00117-8

**Published:** 2017-03-09

**Authors:** Young Ae Cho, Jeonghee Lee, Jae Hwan Oh, Hee Jin Chang, Dae Kyung Sohn, Aesun Shin, Jeongseon Kim

**Affiliations:** 1 Molecular Epidemiology Branch, National Cancer Center, Goyang, South Korea; 2 Center for Colorectal Cancer, National Cancer Center Hospital, National Cancer Center, Goyang, South Korea; 3 Department of Preventive Medicine, Seoul National University College of Medicine, Seoul, South Korea

## Abstract

The role of dietary flavonoid intake in colorectal carcinogenesis might differ according to flavonoid subclasses and individual genetic variants related to carcinogen metabolism. Therefore, we examined whether greater dietary intake of flavonoid subclasses was associated with a lower risk of colorectal cancer and whether *CYP1A1* genetic variants altered this association. A semi-quantitative food frequency questionnaire was used to assess the dietary intake of six flavonoid subclasses (flavonols, flavones, flavanones, flavan-3-ols, anthocyanidins, and isoflavones) in 923 patients with colorectal cancer and 1,846 controls; furthermore, *CYP1A1* genetic variants (rs4646903 and rs1048943) were genotyped. Among the subclasses of flavonoids, higher intake of flavonols and flavan-3-ols showed a stronger association with a reduced risk of colorectal cancer after adjusting for potential confounding factors. Carriers of the *CYP1A1* rs4646903 CC homozygous variant showed a reduced risk of rectal cancer compared with that in TT carriers. The inverse association between dietary flavonol intake and colorectal cancer risk was stronger among carriers of the CC homozygous variant than among T allele carriers (*P* for interaction = 0.02), particularly for rectal cancer (*P* for interaction = 0.005). In conclusion, the effect of dietary flavonoid intake on colorectal cancer risk differs according to flavonoid subclasses and *CYP1A1* genetic variants.

## Introduction

The incidence of colorectal cancer has increased rapidly in Korea in recent decades, potentially due in part to changes in diet and lifestyle^1, 2^. Flavonoids are bioactive polyphenolic compounds that naturally occur in plant-based foods (e.g., fruits, vegetables, grains, and herbs) and drinks (e.g., tea, wine and juices)^3^. Flavonoids are subclassified into flavonols, flavones, flavanones, flavan-3-ols, anthocyanidins, and isoflavones, based on their structure^3^. The various combinations of multiple hydroxyl and methoxyl group substituents on the basic flavonoid skeleton result in numerous compounds with different functions^4^. Based on experimental studies, flavonoids block or suppress multistage carcinogenesis through several biological mechanisms, including antioxidant action, anti-inflammatory activity, and effects on xenobiotic and carcinogen metabolism^3^. However, several epidemiological studies have reported inconsistent findings^5, 6^. An Italian case-control study identified an inverse association between colorectal cancer risk and flavonoids, particularly for certain flavonoid subclasses^5^; however, a US prospective cohort study did not observe this association^6^.

Both environmental and genetic factors likely play important roles in colorectal carcinogenesis. Cytochrome P450 (CYP) family enzymes are involved in the metabolism and detoxification of numerous xenobiotics; thus, the modulation of this enzyme system can influence xenobiotic metabolism^4^. Dietary flavonoids may induce the expression of several CYPs, and modulate CYP metabolic activity. Conversely, some CYPs participate in flavonoid metabolism^4^. Among the genetic variants in *CYPs*, the associations of the *CYP1A1* polymorphisms rs4646903 T > C in the 3′-flanking region and rs1048943 A > G in exon 7 with colorectal cancer has been widely investigated^7, 8^. These polymorphisms may modify CYP1A1 enzyme activity and ultimately affect susceptibility to colorectal cancer^8^. Therefore, we hypothesized that the effect of certain dietary flavonoid subclasses on colorectal carcinogenesis may differ according to the variants in the *CYP1A1* gene.

In this study, we examined whether a greater habitual dietary intake of flavonoid subclasses (flavonols, flavones, flavanones, flavan-3-ols, anthocyanidins, and isoflavones) is associated with a lower risk of colorectal cancer and whether the associations with certain flavonoid subclasses are altered by variants in the *CYP1A1* gene.

## Results

### General characteristics of the study population

The distribution of the characteristics of the controls and cases is shown in Table 1. Significant differences were observed between the cases and controls in terms of the socio-demographic factors and lifestyle habits; cases were more likely to have a family history of colorectal cancer (*P* < 0.001), had a lower level of education (*P* < 0.001), and did not exercise regularly (*P* < 0.001) compared with the controls. The mean of total caloric intake was 1,689.6 kcal/day for the controls and 2,026.3 kcal/day for the cases (*P* < 0.001). Smoking status and alcohol consumption were not different between the cases and controls. The median total dietary flavonoid intake was 98.6 mg/day and 80.1 mg/day for the controls and cases, respectively (*P* < 0.001). With the exception of flavanones, the intake of the five flavonoid subclasses was significantly higher among the controls than among the cases (*P* < 0.001).

**Table 1:** Participants’ general characteristics.

	Controls (n = 1846)	Cases (n = 923)	*P*-value^**^
Age (years)	56.1 ± 9.1	56.6 ± 9.7	0.20
Female	596 (32.3)	(32.3)	>0.99
Family history of colorectal cancer (yes)	99 (5.4)	86 (9.3)	<0.001
BMI (kg/m^2^)			
<25	1226 (66.4)	640 (69.3)	0.12
≥25	620 (33.6)	283 (30.7)	
Educational level			
Middle school or less	282 (15.6)	321 (34.8)	<0.001
High school	587 (32.6)	369 (40.0)	
College or more	934 (51.8)	233 (25.2)	
Smoking status			
Never	818 (44.3)	409 (44.3)	>0.99
Ever	1028 (55.7)	514 (55.7)	
Alcohol consumption			
Never	560 (30.3)	279 (30.2)	0.95
Ever	1286 (69.7)	644 (69.8)	
Regular exercise (yes)	1047 (58.2)	311 (33.7)	<0.001
Total caloric intake (kcal/day)	1689.6 ± 560.4	2026.3 ± 534.0	<0.001
Dietary flavonoids (mg/day)^*^			
Total flavonoids^*^	98.6 (68.3, 141.8)	80.1 (57.5, 106.5)	<0.001
Flavonols^*^	19.2 (13.0, 27.4)	15.0 (11.2, 19.2)	<0.001
Flavones^*^	1.1 (0.7, 1.7)	1.0 (0.8, 1.4)	<0.001
Flavanones^*^	3.6 (1.2, 9.0)	3.7 (1.3, 7.8)	0.13
Flavan-3-ols^*^	13.1 (5.6, 30.4)	8.8 (3.9, 18.6)	<0.001
Anthocyanidins^*^	18.9 (11.4, 29.7)	16.1 (10.7, 24.1)	<0.001
Isoflavones^*^	26.7 (16.1, 43.0)	24.1 (16.2, 34.6)	<0.001

The data are presented as n (%) for the categorical variables, means ± standard deviations (SD) for the continuous variables, and medians (interquartile ranges) for dietary flavonoid intake. *The dietary flavonoid intake and intake of its subclasses were adjusted for total energy intake using the residual method. **P-value was calculated using the χ^2^ test for the categorical variables, t-test for continuous variables, and Wilcoxon rank-sum test for flavonoid variables.

### Association between dietary flavonoid intake and colorectal cancer risk

When comparing the highest and lowest quartiles of flavonoid intake with regard to colorectal cancer risk, significant associations were observed between total flavonoid intake and colorectal cancer risk in both crude and multivariable models adjusted for age, sex, body mass index (BMI), education, total caloric intake, family history of colorectal cancer and regular exercise. With the exception of flavanones, all subclasses showed a significant inverse association between the highest quartile and colorectal cancer risk compared with the lowest quartile. Among them, flavonols (odds ratio (OR) [95% confidence interval (CI)] = 0.18 [0.13, 0.25], highest vs. lowest quartile, *P* for trend < 0.001) and flavan-3-ols (OR [95% CI] = 0.49 [0.38, 0.66], highest vs. lowest quartile, *P* for trend < 0.001) showed a stronger association (Table 2).

**Table 2:** Association between dietary flavonoid intake and the risk of colorectal cancer.

Dietary flavonoids (mg/day)	No. of Controls (%)	No. of Cases (%)	Crude OR (95% CI)	Multivariable OR (95% CI)
*Total Flavonoids*				
Q1 (<67.7)	461 (25.0)	347 (37.6)	1.0 (ref)	1.0 (ref)
Q2 (67.7–<98.3)	462 (25.0)	295 (32.0)	0.85 (0.69‒1.04)	0.91 (0.72, 1.14)
Q3 (98.3–<141.7)	462 (25.0)	212 (23.0)	0.61 (0.49‒0.76)	0.66 (0.52, 0.85)
Q4 (≥141.7)	461 (25.0)	69 (7.5)	0.20 (0.15‒0.27)	0.20 (0.14, 0.28)
*P* for trend			<0.001	<0.001
*Flavonols*				
Q1 (<13.0)	461 (25.0)	337 (36.5)	1.0 (ref)	1.0 (ref)
Q2 (13.0–<19.2)	462 (25.0)	355 (38.5)	1.05 (0.86‒1.28)	1.05 (0.84, 1.32)
Q3 (19.2–<27.4)	462 (25.0)	163 (17.7)	0.48 (0.39‒0.61)	0.50 (0.39, 0.65)
Q4 (≥27.4)	461 (25.0)	68 (7.4)	0.20 (0.15‒0.27)	0.18 (0.13, 0.25)
*P* for trend			<0.001	<0.001
*Flavones*				
Q1 (<0.75)	461 (25.0)	213 (23.1)	1.0 (ref)	1.0 (ref)
Q2 (0.75–<1.12)	462 (25.0)	306 (33.2)	1.43 (1.15, 1.78)	1.50 (1.17, 1.92)
Q3 (1.12–<1.70)	462 (25.0)	292 (31.6)	1.37 (1.10, 1.70)	1.34 (1.04, 1.73)
Q4 (≥1.70)	461 (25.0)	112 (12.1)	0.53 (0.40, 0.68)	0.49 (0.36, 0.67)
*P* for trend			<0.001	<0.001
*Flavanones*				
Q1 (<1.16)	462 (25.0)	236 (25.6)	1.0 (ref)	1.0 (ref)
Q2 (1.16–<3.54)	461 (25.0)	228 (24.7)	0.97 (0.78, 1.21)	1.16 (0.90, 1.50)
Q3 (3.54–<8.99)	462 (25.0)	272 (29.5)	1.15 (0.93, 1.43)	1.37 (1.07, 1.76)
Q4 (≥8.99)	461 (25.0)	187 (20.3)	0.79 (0.63, 1.00)	0.97 (0.74, 1.27)
*P* for trend			0.03	0.40
*Flavan-3-ols*				
Q1 (5.62)	462 (25.0)	330 (35.8)	1.0 (ref)	1.0 (ref)
Q2 (5.62–<13.19)	461 (25.0)	272 (29.5)	0.83 (0.67, 1.02)	0.90 (0.71, 1.14)
Q3 (13.19–<30.38)	462 (25.0)	196 (21.2)	0.59 (0.48, 1.74)	0.67 (0.52, 0.87)
Q4 (≥30.38)	461 (25.0)	125 (13.5)	0.38 (0.30, 0.48)	0.49 (0.38, 0.66)
*P* for trend			<0.001	<0.001
*Anthocyanidins*				
Q1 (<11.4)	461 (25.0)	260 (28.2)	1.0 (ref)	1.0 (ref)
Q2 (11.4-<18.9)	462 (25.0)	286 (31.0)	1.10 (0.89, 1.36)	1.22 (0.96, 1.56)
Q3 (18.9–<29.7)	462 (25.0)	243 (26.3)	0.93 (0.75, 1.16)	0.99 (0.77, 1.26)
Q4 (≥29.7)	461 (25.0)	134 (14.5)	0.52 (0.40, 0.66)	0.54 (0.40, 0.71)
*P* for trend			<0.001	<0.001
*Isoflavones*				
Q1 (16.2)	462 (25.0)	224 (24.3)	1.0 (ref)	1.0 (ref)
Q2 (16.2–<26.7)	461 (25.0)	322 (34.9)	1.44 (1.16, 1.78)	1.28 (1.01, 1.64)
Q3 (26.7–<43.0)	461 (25.0)	234 (25.4)	1.05 (0.84, 1.31)	1.01 (0.78, 1.30)
Q4 (≥43.0)	462 (25.0)	143 (15.5)	0.64 (0.50, 0.82)	0.61 (0.46, 0.81)
*P* for trend			<0.001	<0.001

*Multivariable OR was adjusted for age, sex, BMI, education, total caloric intake, family history of colorectal cancer, and regular exercise.

The association between dietary flavonoid intake and colorectal cancer risk did not differ by anatomic site (Supplementary Table S1). However, in the analysis stratified by sex, an inverse relationship between isoflavone intake and colorectal cancer was observed among men (OR [95% CI] = 0.50 [0.35, 0.72], highest vs. lowest quartile, *P* for trend < 0.001), but not women (OR [95% CI] = 0.87 [0.54, 1.41], highest vs. lowest quartile, *P* for trend = 0.34) (Supplementary Table S2).

### Association between *CYP1A1* genetic variants and colorectal cancer risk

The *CYP1A1* rs4646903 and rs1048943 variants have minor allele frequency of 0.39 and 0.24, respectively. These polymorphisms were in Hardy-Weinberg equilibrium (HWE) among the controls and were not associated with colorectal cancer risk. However, when the data were stratified by the anatomic site, homozygous variant of *CYP1A1* rs4646903 showed an inverse association with the risk of rectal cancer (OR [95% CI] = 0.64 [0.42, 0.98], CC vs. TT). However, no association was observed with rs1048943 (Table 3).

**Table 3:** Association between the *CYP1A1* genetic variants and the risks of colorectal cancer, colon cancer, and rectal cancer.

*CYP1A1* Genetic Variants	No. of Controls (%)	Colorectal Cancer			Colon Cancer			Rectal Cancer		
		No. of Cases (%)	Crude OR (95% CI)	Multivariable OR (95% CI)^*^	No. of Cases (%)	Crude OR (95% CI)	Multivariable OR (95% CI)^*^	No. of Cases (%)	Crude OR (95% CI)	Multivariable OR (95% CI)^*^
***rs4646903***										
TT	525 (37.5)	268 (38.5)	1.0 (ref)	1.0 (ref)	130 (37.1)	1.0 (ref)	1.0 (ref)	133 (39.8)	1.0 (ref)	1.0 (ref)
TC	646 (46.1)	323 (46.3)	0.97 (0.80, 1.18)	0.96 (0.77, 1.21)	156 (44.6)	0.95 (0.73, 1.23)	0.94 (0.72, 1.24)	161 (48.2)	0.99 (0.76, 1.28)	0.98 (0.75, 1.31)
CC	229 (16.4)	106 (15.2)	0.90 (0.68, 1.18)	0.83 (0.61, 1.26)	64 (18.3)	1.10 (0.79, 1.54)	1.04 (0.72, 1.49)	40 (12.0)	0.69 (0.47, 1.02)	0.64 (0.42, 0.98)
***rs1048943***										
AA	804 (58.6)	422 (60.7)	1.0 (ref)	1.0 (ref)	210 (60.0)	1.0 (ref)	1.0 (ref)	205 (61.8)	1.0 (ref)	1.0 (ref)
AG	483 (35.2)	237 (34.1)	0.96 (0.79, 1.16)	0.95 (0.76, 1.18)	119 (34.0)	0.96 (0.75, 1.24)	0.95 (0.72, 1.24)	114 (34.3)	0.95 (0.74, 1.23)	0.93 (0.70, 1.22)
GG	84 (6.1)	36 (5.2)	0.84 (0.56, 1.26)	0.81 (0.51, 1.28)	21 (6.0)	0.62 (0.34, 1.14)	0.98 (0.57, 1.68)	13 (3.9)	0.62 (0.34, 1.14)	0.62 (0.32, 1.18)

*The multivariable OR was adjusted for age, sex, BMI, education, total caloric intake, family history of colorectal cancer, and regular exercise.

### Interaction between *CYP1A1* rs4646903 and flavonols/flavan3-ols regarding colorectal cancer risk

The inverse association between flavonol intake and colorectal cancer risk was stronger among carriers of the rs4646903 CC homozygous variant than among T allele carriers (OR [95% CI] = 0.19 [0.11‒0.33], CC carriers with high flavonol intake vs. TT/TC carriers with low flavonol intake, *P* for interaction = 0.02). However, flavan-3-ols did not show any interaction regarding colorectal cancer risk. Therefore, further stratified analyses were performed only for flavonols. When stratified by anatomic site, the interaction between flavonols and rs4646903 variant was stronger for rectal cancer (OR [95% CI] = 0.09 [0.03‒0.25], CC carriers with high flavonol intake vs. TT/TC carriers with low flavonol intake, *P* for interaction = 0.005) (Table 4). In addition, in the stratified analysis of other risk factors, the interaction between flavonols and *CYP1A1* rs4646903 in relation to colorectal cancer risk was stronger among individuals older than 50 years (*P* for interaction = 0.03) and individuals who did not exercise regularly (*P* for interaction = 0.03), smoked (*P* for interaction = 0.006), or drank alcoholic beverages (*P* for interaction = 0.02) (Supplementary Table S3).

**Table 4:** Combined effect of dietary flavonol intake and the *CYP1A1* rs4646903 variant on the risks of colorectal cancer, colon cancer, and rectal cancer.

Dietary flavonol intake	*CYP1A1* rs4646903				*P* for interaction
	TT/TC		CC		
	Low	High	Low	High	
Colorectal cancer					
No. Controls/Cases	602/439	569/152	99/88	130/18	0.02
OR (95% CI)^*^	1.0 (ref)	0.38 (0.29, 0.48)	1.08 (0.76, 1.54)	0.19 (0.11, 0.33)	
Colon cancer					
No. Controls/Cases	602/213	569/73	99/50	130/14	0.24
OR (95% CI)^*^	1.0 (ref)	0.36 (0.26, 0.49)	1.28 (0.85, 1.91)	0.28 (0.15, 0.52)	
Rectal cancer					
No. Controls/Cases	602/217	569/77	99/36	130/4	0.005
OR (95% CI)^*^	1.0 (ref)	0.40 (0.29, 0.554)	0.88 (0.57, 1.38)	0.09 (0.03, 0.25)	

Dietary flavonol intake was categorized into two groups based on the median level in the control group. *The multivariable OR was adjusted for age, sex, BMI, education, total caloric intake, family history of colorectal cancer, and regular exercise.

## Discussion

As shown in the present study, dietary flavonoid intake, particularly flavonols and flavan-3-ols, was associated with a decreased risk of colorectal cancer. In addition, *CYP1A1* rs4646903 genetic variant appears to modify the roles of flavonols in colorectal carcinogenesis. CC homozygous carriers with higher flavonol intake had substantially lower rates of colorectal cancer, particularly rectal cancer.

Several previous studies identified different associations based on the flavonoid subclass, consistent with the results of the present study. As shown in an Italian case-control study, individuals who consumed higher levels of isoflavones, anthocyanidins, flavones and flavonols had a reduced risk of colorectal cancer, but an association with catechin or flavanone intake was not observed^9^. According to a large Scottish case-control study, higher intake of flavonols and catechins, but not flavanones or phyto-estrogens, was associated with a reduced colorectal cancer risk^10^. In the present study, we observed an inverse association for the five subclasses (flavonols, flavones, flavan-3-ols, anthocyanidins, and isoflavones) in a multivariable model that included potential confounders. Among the investigated flavonoids, flavonols and flavan-3-ols showed a stronger association with a reduced risk of colorectal cancer. Flavan-3-ols and flavonols inhibit the growth of human colon cancer cell lines *in vitro* ^11^. In addition, flavan-3-ols can reactivate silenced tumor suppressor genes in colorectal cancer cells^12^. Moreover, a secondary analysis of the Polyp Prevention Trial revealed that flavonol intake was associated with a lower risk of advanced adenoma recurrence^13^. However, two large prospective cohort studies did not show an association between the habitual intake of any flavonoid subclass and the risk of colorectal cancer^6^. The different associations reported in each study might be due to the differences in study design and the distribution of the flavonoid subclasses, as well as to variations in flavonoid intake levels among different populations.

The underlying mechanisms by which flavonols and flavan-3-ols exert stronger protective effects require further investigation. Flavonoid subclasses exhibit large differences in bioavailability and might have diverse beneficial effects^14^. Differences in chemical structure might endow the subclasses with various abilities, such as scavenging reactive oxygen species or suppressing cellular proliferation^15^. Among the individual flavonoids tested in experimental studies, only quercetin suppressed the proliferation of colon cancer cell lines and modulated cell growth signaling pathways^16^. Substitutions with hydroxyl groups at various positions determine whether flavonoids induce or inhibit *CYP1A1* expression under different experimental conditions^16^. In addition, flavonoid metabolism might affect its biological function. Because flavonoids are rarely bioavailable, a large fraction of dietary flavonoids remain in the large intestine^17^. Intestinal microflora can metabolize these flavonoids, and some of these metabolites might exhibit different biological activities than their parent compounds^17^. Therefore, inter-individual variations in the bacterial strains in the gut, and thus different abilities to metabolize flavonoids, might increase the complexity of the underlying mechanism of action of flavonoids^18^.

The role of CYPs in carcinogenesis remains controversial. Because CYPs are involved in activating procarcinogens, strategies that inhibit CYP activity might be beneficial for preventing colorectal cancer^3^. However, CYPs are also involved in detoxification processes by regulating phase II enzymes (e.g., glutathinone S-transferase and N-acetyltransferase)^19^. Therefore, in this situation, the up-regulation of *CYP* genes might be protective against carcinogenesis. As shown in the present study, the rs4646903 variant may be associated with a reduced risk of colorectal cancer; thus, *CYP1A1* may be involved in detoxification. Cleary *et al.* ^20^ also observed an association between the homozygous *CYP1A1* variant and a decreased risk of colorectal cancer. *CYP1A1* rs4646903 variant may be associated with a greater induction of enzyme activity^21^, which is associated with colorectal cancer^8^. Ethnic differences in the allele frequency of *CYP1A1* rs4646903 have yielded different associations among studies; this particular allele frequency is higher in East Asians, including Koreans, than in other races^22^. In this study, the *CYP1A1* genetic variant was suggested to play a role in the etiology of colorectal cancer. This role of the *CYP1A1* genetic variant may be affected by the combined influence of other genes involved in metabolizing carcinogens and alterations in exposure to environmental factors, such as flavonoids^19^.

*CYP1A1* might interact with flavonoid compounds in several ways^4^. First, flavonoids induce CYP expression and modulate their enzymatic activity^4, 16^. Some flavonoids alter CYP activity by binding to the aryl hydrocarbon receptor (AhR), a ligand-activated transcription factor, as either AhR agonists or antagonists. In addition, the activation of phase II detoxification of carcinogens might be associated with the anti-carcinogenic effects of flavonoids^3^. Second, flavonoids are metabolized by CYP1A1^4^, and CYP1A1 activity is associated with colorectal carcinogenesis^8, 23^. Therefore, *CYP1A1* genetic variants can alter flavonoid metabolism and subsequently modify the association between flavonoids and the risk of cancer. Therefore, variations in CYP1A1 activity in response to dietary flavonoids and genetic variations might affect carcinogen metabolism^16^. As shown in the present study, the protective effect of flavonols is stronger among *CYP1A1* rs4646903 CC homozygous carriers than among T allele carriers. Based on this result, both genetic variants and flavonols may be involved in the detoxification of carcinogens^3^. Although the agonist function of flavonoids does not correspond with their anti-cancer properties, other mechanisms such as the induction of phase II enzymes, the suppression of cell cycle progression and the inhibition of several CYP enzyme activities might help detoxify carcinogens^3, 4^.

Because colorectal cancer is a heterogeneous disease^24^, the effects of genetic variants and dietary factors may differ according to the anatomic site. In the present study, the rs4646903 genetic variant was inversely associated with rectal cancer, but not colon cancer. Additionally, a stronger interaction between flavonol intake and rs4646903 genetic variant was also observed only for rectal cancer. Because the colon and rectum differ in terms of metabolic enzyme activity, physiological function, fecal composition, bile acid metabolism, and intestinal transit time, environmental factors and genetic variants regulating detoxifying enzymes may have different effects according to the anatomic location^24^. One cohort study of women also reported an inverse association with catechin intake that was confined to rectal cancer^25^. However, the evidence is still limited, precluding hypotheses about any mechanisms; thus, further studies are required.

In addition, several other factors might affect the observed associations. First, the association of isoflavone intake with colorectal cancer seems to be sex-specific. A significant association was only observed among men, similar to findings from a Japanese case-control study^26^. Because isoflavones have a diphenolic structure similar to 17β-estradiol, it exerts an estrogen-like effect by binding to estrogen receptors^27^. Isoflavones have been reported to exert a protective effect against colorectal cancer only in an environment with a low estrogen level^27^. We also expected to observe sex-specific differences in the interactions because the activities of CYP1A1 and the flavonoid-mediated alterations of this enzyme can differ by sex^28^. However, sex-specific differences were not observed in the current study. Second, compared with younger people, older people showed a stronger interaction between *CYP1A1* genetic variants and flavonol intake with regard to colorectal cancer risk, possibly because of a greater effect of the environmental factors. Third, unhealthy lifestyle habits, such as physical inactivity, smoking and alcohol consumption, might increase the interaction between flavonol intake and *CYP1A1* polymorphisms with regard to colorectal cancer risk. Finally, the effects of flavonoids on CYPs depend on their structure and concentration as well as the tissue distribution of the enzymes^4, 16^. Thus, inter-individual variability in carcinogen-metabolizing enzymes caused by genetic and environmental factors might contribute to the differences in the susceptibility of different individuals to colorectal cancer.

Several limitations should be considered when interpreting our findings. First, this study applied a case-control design; thus both recall and selection bias might be present. Because flavonoids are consumed by ingesting plant-based foods and beverages, patients with colorectal cancer might have a differential recall of these foods compared with healthy control participants^6^. Second, the semi-quantitative food frequency questionnaire (SQFFQ) was not specifically designed to investigate flavonoid intake. Therefore, certain foods rich in flavonoids (e.g., herbs) are missing from this survey. Moreover, flavonoid content can vary depending on species, seasonal variation, food processing, and storage^29^. However, the data were collected using a validated questionnaire and the participants were not aware of the specific hypotheses of the current study, thus reducing the potential for differential misclassification. Third, the amount of flavonoid intake in the present study was relatively small compared with that in another Korean study using a different flavonoid database^30^, although it is similar to a study using the same database^31^. The flavonoid database used in the present study did not include proanthocyanidins, which had the greatest contribution to total flavonoid intake. In addition, the thearubigin content in this database is a crude estimation quantified using an indirect method^32^. However, the inclusion of thearubigins may not seriously affect the estimated flavonoid intake because tea, the only food source of thearubigins, is not consumed in large quantities by Koreans. A more comprehensive flavonoid database is needed to improve the quality of this study. Finally, the sample size used for the stratified analyses of the gene-diet interaction was relatively small, and thus the findings may not be very robust.

In conclusion, the effect of dietary flavonoid intake on colorectal cancer risk may differ by subclass and individual genetic variants. Individuals with different *CYP1A1* variants might experience a different benefit from the intake of certain dietary flavonoid subclasses on the prevention of colorectal cancer. Furthermore, an improved understanding of the interactions between flavonoid intake and the genetic variants might provide insights into the mechanisms of carcinogenesis and assist with the development of strategies to prevent colorectal cancer. However, larger studies are needed to validate the findings.

## Methods

### Study population

Cases were patients who were newly diagnosed with colorectal cancer (between August 2010 and August 2013) at the Center for Colorectal Cancer within the National Cancer Center in Korea. Of the 1,070 patients who agreed to participate in this study and provided informed consent, 145 were excluded because they provided incomplete SQFFQs and two were removed because of implausible energy intakes (<500 kcal/day or >4,000 kcal/day). Therefore, 923 patients were included in the analysis. The controls were selected from a group of participants who visited the Center for Cancer Prevention and Detection at the same hospital between October 2007 and December 2014 for a health check-up program provided by the National Health Insurance Cooperation, which covers the entire Korean population. Of the 14,201 participants who agreed to participate in this study, 5,044 were excluded because they provided incomplete SQFFQs (n = 4,779) and questionnaires (n = 265), and 120 were excluded because of implausible energy intakes. Of the remaining 9,037 participants, two controls per case were randomly selected according to gender and the 5-year age group. The randomization procedure was accomplished using SAS statistical software (SAS Institute, Cary, NC) using the Surveyselect procedure to implement a random sampling within strata of gender and the 5-year age group. Therefore, 923 cases and 1,846 controls were selected to analyze the association between dietary flavonoid intake and colorectal cancer risk. Individuals for whom a blood sample was not available were excluded from the investigation of the association between the genetic variants and their interactions with flavonoids regarding the risk of colorectal cancer. Therefore, 701 patients with colorectal cancer and 1,402 healthy controls were selected (Supplementary Fig. S1).

All the participants provided written informed consent prior to participation, and the Institutional Review Board (IRB) of the National Cancer Center approved the study protocols (IRB Nos NCCNCS-10-350 and NCC2015-0202). All actual procedures utilized in present study were performed in accordance with the guidelines and regulations of the IRB of the National Cancer Center.

### Data collection

A trained interviewer conducted a face-to-face interview to collect information on lifestyle factors and dietary habits prior to the cancer diagnosis. Information regarding the participants’ demographic and lifestyle risk factors (e.g., smoking, alcohol drinking, and regular exercise) was collected using a structured questionnaire. Each participant’s habitual dietary intake was assessed using a 106-item SQFFQ. The validity and reproducibility of this questionnaire were reported previously^33^.

Individual food intake was calculated using CAN-PRO 4.0 (Computer Aided Nutritional Analysis Program, The Korean Nutrition Society, Seoul, Korea). The intake of flavonols, flavones, flavanones, flavan-3-ols, anthocyanidins, and isoflavones was estimated based on the flavonoid database developed by Yang *et al.* ^34^. Briefly, the flavonoid database was constructed using the United States Department of Agriculture (USDA) flavonoid database, the Korea functional food composition table, the Japan functional food factor database, and an additional literature search. This database contains values for thirty individual flavonoids in six flavonoid subclasses expressed as aglycone forms. The intake of each flavonoid subclass was calculated by summing the following individual flavonoids: (1) flavonols: isorhamnetin, kaempferol, myricetin, and quercetin; (2) flavones: apigenin and luteolin; (3) flavanones: eriodictyol, hesperetin, and naringenin; (4) flavan-3-ols: catechin, epigallocatechin, epicatechin, epicatechin 3-gallate, epigallocatechin 3-gallate, gallocatechin, catechin 3-gallate theaflavin, thearubigins, theaflavin-3,3-digallate, theaflavin-3-gallate, and theaflavin-3′-gallate; (5) anthocyanidins: cyanidin, delphinidin, malvidin, pelargonidin, peonidin, and petunidin; and (6) isoflavones: daidzein, genistein, and glycitein. Total flavonoid intake was calculated as the sum of the above flavonoid subclasses. In the present study, the main sources of flavonols were onions and radish; flavones were obtained from hot pepper and kumquat; flavanones were obtained from tangerine, orange, kumquat, and fruit juice; flavan-3-ols were primarily obtained from green tea, grape, pear, and apple; anthocyanidins were obtained from radish, cabbage, persimmon, pear, and kimchi; and isoflavones were obtained from soybean and tofu. The validity of the FFQ for flavonoid intake has been tested in 202 people using the 3-day dietary record as a gold standard^35^. The crude and de-attenuated energy-adjusted correlation coefficients for total flavonoids were 0.36 and 0.32, respectively.

### Genotyping

The *CYP1A1* polymorphisms (rs4646903 T > C and rs1048943 A > G) were genotyped as described below. Genomic DNA was extracted using the MagAttract DNA Blood M48 kit (Qiagen, Hilden, Germany) and BioRobot M48 automatic extraction equipment (Qiagen), according to the manufacturer’s instructions. Genotyping was performed using the MassARRAY iPLEX Gold Assay (Agena Bioscience, San Diego, CA). We included duplicate samples for 3% of the subjects in our initial genotyping analysis, and the rate of discordance was <1%. Finally, genotyping was successfully performed for 697 cases and 1,400 controls for rs4646903 and for 695 cases and 1,371 controls for rs1048943.

### Statistical analyses

The differences in the demographic and lifestyle factors between the cases and controls were analyzed using the χ^2^ test for categorical variables, t-test for continuous variables, and Wilcoxon rank-sum test for dietary intake of flavonoids. The intake of total flavonoids and their subclasses was adjusted for total energy intake using the regression residual method^36^.

The intake levels of dietary flavonoids were categorized into quartiles based on the distribution of the control group to investigate the association between dietary flavonoid intake and colorectal cancer risk. The lowest quartile of each dietary flavonoid intake was used as the reference category. ORs and 95% CIs were estimated using unconditional logistic regression models. Multivariable model was adjusted for age, sex, BMI, education, a family history of colorectal cancer (first-degree relative), regular exercise, and total caloric intake. The median intake of each quartile of flavonoid intake was used as a continuous variable to test for trends. Stratified analyses were performed according to the anatomic location and sex. A polytomous logistic regression model was used for the analyses stratified by anatomic location (colon and rectal cancer).

The χ^2^ test was used to test for HWE of *CYP1A1* rs4646903 and rs1048943 in the control group. We examined the relationship between flavonol and flavan-3-ol intake with colorectal cancer risk based on the *CYP1A1* rs4646903 genetic variant to investigate a gene-diet interaction; the flavonoid subclass and genetic variant were chosen based on the strength and robustness of the associations and the power of the study. Dietary intake of flavonols and flavan-3-ols was categorized into two levels (high/low) based on the median intake levels in the control group. Then, we examined the combined effect of both *CYP1A1* rs4646903 genetic variant and flavonoid subclass intakes (flavonol/flavan-3-ol). Interactions were assessed using the likelihood ratio test by comparing the model including the interaction term with the model that only contained the main effects.

In addition, we conducted stratified analyses to evaluate potential modulatory effects on the interactions between rs4646903 genetic variant and flavonol intake in relation to colorectal cancer risk. We stratified the data according to the following colorectal cancer risk factors: age group (<50 years old and ≥50 years old), sex, BMI (<25 kg/m^2^ and ≥25 kg/m^2^), regular exercise (yes/no), alcohol intake (never/ever), and smoking status (never/ever).

Power analyses were conducted using Quanto 1.2.4^37^. This study has appropriate power to examine the gene-diet interaction as well as main effects. For the gene-diet interaction, the power calculation showed a power of 0.81 to 0.99 to detect an interaction effect between 0.15 to 0.35 for the rs4646903 recessive model, based on our assumption (genetic OR = 0.9 and environmental OR = 0.5). All the statistical analyses were performed using SAS 9.2 (SAS Institute Inc., Cary, NC). A two-sided *P*-value of less than 0.05 was considered statistically significant.

## Electronic supplementary material

Supplementary Information
