# Supplementary material for: Dietary Flavonoids, CYP1A1 Genetic Variants, and the Risk of Colorectal Cancer in a Korean population
Source: Sci Rep. 2017 Mar 9;7:128. doi: 10.1038/s41598-017-00117-8 (PMC5427897; doi:10.1038/s41598-017-00117-8)
Supplement: Supplementary file 1 — Supplementary Information [file 41598_2017_117_MOESM1_ESM.pdf]

Dietary Flavonoids, *CYP1A1* Genetic Variants, and the Risk of Colorectal Cancer in a Korean population

Young Ae Cho[1], Jeonghee Lee[1], Jae Hwan Oh[2], Hee Jin Chang[2], Dae Kyung Sohn[2], Aesun Shin[1,3*] & Jeongseon Kim[1*]

[1]Molecular Epidemiology Branch, National Cancer Center, Goyang, South Korea. [2]Center for Colorectal Cancer, National Cancer Center Hospital, National Cancer Center, South Korea. [3]Department of Preventive Medicine, Seoul National University College of Medicine, Seoul, South Korea

*Corresponding Authors: Jeongseon Kim (e-mail: jskim@ncc.re.kr); Aesun Shin (e-mail: shinaesun@snu.ac.kr)

Supplementary Table S1. Association between dietary flavonoid intake and the risk of colorectal cancer according to anatomic site. Multivariable OR was adjusted for age, sex, BMI, education, total caloric intake, family history of colorectal cancer, and regular exercise.

**Dietary flavonoids (mg/day)**	**No. of Controls (%)**	**Colon cancer**		**Rectal cancer**	
		**No. of Cases (%)**	**Multivariable OR (95% CI)**	**No. of Cases (%)**	**Multivariable OR (95% CI)**
Total Flavonoids					
Q1 (<67.7)	461 (25.0)	163 (35.4)	1.0 (ref)	172 (38.7)	1.0 (ref)
Q2 (67.7-<98.3)	462 (25.0)	149 (32.4)	0.94 (0.71, 1.24)	143 (32.2)	0.92 (0.69, 1.22)
Q3 (98.3-<141.7)	462 (25.0)	112 (24.4)	0.70 (0.52, 0.95)	96 (21.6)	0.63 (0.46, 0.86)
Q4 (≥141.7)	461 (25.0)	36 (7.8)	0.20 (0.13, 0.31)	33 (7.4)	0.21 (0.14, 0.32)
P for trend			< 0.001		< 0.001
Flavonols					
Q1 (<13.0)	461 (25.0)	160 (34.8)	1.0 (ref)	164 (36.9)	1.0 (ref)
Q2 (13.0-<19.2)	462 (25.0)	182 (39.6)	1.11 (0.85, 1.46)	170 (28.3)	1.06 (0.80, 1.40)
Q3 (19.2-<27.4)	462 (25.0)	84 (18.3)	0.52 (0.38, 0.72)	77 (17.3)	0.51 (0.37, 0.71)
Q4 (≥27.4)	461 (25.0)	34 (7.4)	0.18 (0.12, 0.27)	33 (7.4)	0.19 (0.12, 0.30)
P for trend			< 0.001		< 0.001
Flavones					
Q1 (<0.75)	461 (25.0)	101 (22.0)	1.0 (ref)	106 (23.9)	1.0 (ref)
Q2 (0.75-<1.12)	462 (25.0)	144 (31.3)	1.47 (1.08, 2.01)	155 (34.9)	1.55 (1.14, 2.10)
Q3 (1.12-<1.70)	462 (25.0)	150 (32.6)	1.38 (1.02, 1.89)	138 (31.1)	1.33 (0.97, 1.82)
Q4 (≥1.70)	461 (25.0)	65 (14.1)	0.56 (0.39, 0.82)	45 (10.1)	0.42 (0.28, 0.64)
P for trend			< 0.001		< 0.001
Flavanones					
Q1 (<1.16)	462 (25.0)	110 (23.9)	1.0 (ref)	123 (27.7)	1.0 (ref)
Q2 (1.16-<3.54)	461 (25.0)	110 (23.9)	1.18 (0.86, 1.63)	115 (25.9)	1.16 (0.84, 1.58)
Q3 (3.54-<8.99)	462 (25.0)	146 (31.7)	1.51 (1.11, 2.05)	117 (26.4)	1.20 (0.88, 1.64)
Q4 (≥8.99)	461 (25.0)	94 (20.4)	0.97 (0.69, 1.36)	89 (20.1)	0.96 (0.68, 1.36)
P for trend			0.44		0.50
Flavan-3-ols					
Q1 (5.62)	462 (25.0)	163 (35.4)	1.0 (ref)	160 (36.0)	1.0 (ref)
Q2 (5.62-<13.19)	461 (25.0)	137 (29.8)	0.88 (0.67, 1.18)	128 (28.8)	0.90 (0.67, 1.20)
Q3 (13.19-<30.38)	462 (25.0)	91 (19.8)	0.60 (0.44, 0.82)	102 (23.0)	0.76 (0.56, 1.04)
Q4 (≥30.38)	461 (25.0)	69 (15.0)	0.52 (0.37, 0.73)	54 (12.2)	0.47 (0.33, 0.68)
P for trend			< 0.001		< 0.001
Anthocyanidins					
Q1 (<11.4)	461 (25.0)	117 (25.4)	1.0 (ref)	136 (30.6)	1.0 (ref)
Q2 (11.4-<18.9)	462 (25.0)	142 (30.9)	1.31 (0.97, 1.77)	140 (31.5)	1.18 (0.88, 1.59)
Q3 (18.9-<29.7)	462 (25.0)	131 (28.5)	1.13 (0.83, 1.53)	105 (23.7)	0.84 (0.61, 1.14)
Q4 (≥29.7)	461 (25.0)	70 (15.2)	0.59 (0.41, 0.84)	63 (14.2)	0.51 (0.36, 0.73)
P for trend			< 0.001		< 0.001
Isoflavones					
Q1 (16.2)	462 (25.0)	109 (23.7)	1.0 (ref)	109 (24.6)	1.0 (ref)
Q2 (16.2-<26.7)	461 (25.0)	150 (32.6)	1.23 (0.91, 1.67)	161 (36.3)	1.34 (0.99, 1.81)
Q3 (26.7-<43.0)	461 (25.0)	128 (27.8)	1.10 (0.81, 1.50)	104 (23.4)	0.94 (0.68, 1.30)
Q4 (≥43.0)	462 (25.0)	73 (15.9)	0.62 (0.44, 0.88)	70 (15.8)	0.64 (0.45, 0.91)
P for trend			0.001		< 0.001

Supplementary Table S2. Association between dietary flavonoid intake and the risk of colorectal cancer according to sex. Multivariable OR was adjusted for age, sex, BMI, education, total caloric intake, family history of colorectal cancer, and regular exercise.

	**Men**		**Women**	
**Dietary flavonoids (mg/day)**	**No. of Controls/cases**	**Multivariable OR (95% CI)**	**No. of Controls/cases**	**Multivariable OR (95% CI)**
Total Flavonoids				
Q1 (<67.7)	364/266	1.0 (ref)	97/81	1.0 (ref)
Q2 (67.7-<98.3)	326/204	0.94 (0.72, 1.24)	136/91	0.85 (0.54, 1.33)
Q3 (98.3-<141.7)	311/123	0.57 (0.42, 0.77)	151/89	0.88 (0.56, 1.38)
Q4 (≥141.7)	249/32	0.20 (0.13, 0.31)	212/37	0.22 (0.13, 0.37)
P for trend		< 0.001		< 0.001
Flavonols				
Q1 (<13.0)	355/250	1.0 (ref)	106/87	1.0 (ref)
Q2 (13.0-<19.2)	329/248	1.10 (0.84, 1.43)	133/107	0.96 (0.62, 1.47)
Q3 (19.2-<27.4)	312/94	0.42 (0.31, 0.59)	150/69	0.66 (0.42, 1.04)
Q4 (≥27.4)	254/33	0.17 (0.11, 0.27)	207/35	0.21 (0.12, 0.35)
P for trend		< 0.001		< 0.001
Flavones				
Q1 (<0.75)	372/172	1.0 (ref)	89/41	1.0 (ref)
Q2 (0.75-<1.12)	354/230	1.46 (1.10, 1.95)	108/76	1.68 (0.99, 2.86)
Q3 (1.12-<1.70)	300/165	1.16 (0.86, 1.58)	162/127	1.75 (1.07, 2.86)
Q4 (≥1.70)	224/58	0.56 (0.38, 0.82)	237/54	0.52 (0.31, 0.88)
P for trend		0.002		< 0.001
Flavanones				
Q1 (<1.16)	382/199	1.0 (ref)	80/37	1.0 (ref)
Q2 (1.16-<3.54)	347/170	1.15 (0.86, 1.54)	114/58	1.25 (0.71, 2.21)
Q3 (3.54-<8.99)	313/166	1.17 (0.87, 1.58)	149/106	1.84 (1.08, 3.12)
Q4 (≥8.99)	208/90	1.11 (0.78, 1.57)	253/97	0.96 (0.57, 1.60)
P for trend		0.65		0.10
Flavan-3-ols				
Q1(5.62)	367/250	1.0 (ref)	95/80	1.0 (ref)
Q2 (5.62-<13.19)	325/176	0.84 (0.64, 1.12)	136/96	0.95 (0.61, 1.49)
Q3 (13.19-<30.38)	282/122	0.71 (0.52, 0.97)	180/74	0.57 (0.36, 0.89)
Q4 (≥30.38)	276/77	0.55 (0.39, 0.78)	185/48	0.42 (0.26, 0.69)
P for trend		< 0.001		< 0.001
Anthocyanidins				
Q1 (<11.4)	347/198	1.0 (ref)	114/62	1.0 (ref)
Q2 (11.4-<18.9)	327/202	1.21 (0.90, 1.61)	135/84	1.15 (0.72, 1.83)
Q3 (18.9-<29.7)	317/149	0.84 (0.62, 1.14)	146/94	1.33 (0.84, 2.10)
Q4 (≥29.7)	259/76	0.55 (0.38, 0.79)	202/58	0.54 (0.33, 0.87)
P for trend		< 0.001		0.002
Isoflavones				
Q1 (16.2)	320/160	1.0 (ref)	142/64	1.0 (ref)
Q2 (16.2-<26.7)	307/232	1.22 (0.90, 1.64)	154/90	1.33 (0.85, 2.07)
Q3 (26.7-<43.0)	313/147	0.84 (0.61, 1.15)	148/87	1.47 (0.94, 2.30)
Q4 (≥43.0)	310/86	0.50 (0.35, 0.72)	152/57	0.87 (0.54, 1.41)
P for trend		< 0.001		0.34

Supplementary Table S3. Effects of dietary flavonol intake on the risk of colorectal cancer stratified by *CYP1A1* rs4646903 variants and risk factors. *The flavonol intake was categorized into two groups (high/low) based on the median (19.1 mg/day) level of the control group. **Multivariable OR was adjusted for age, sex, BMI, education, total caloric intake, family history of colorectal cancer, and regular exercise.

	***CYP1A1* rs4646903**						
	**TT/TC**			**CC**			**_P_ for interaction**
**Risk Factors**	**No. Controls/Cases**		**High vs. Low***	**No. Controls/Cases**		**High vs. Low***	
	**Low***	**High***	**Multivariable OR (95% CI)****	**Low***	**High***	**Multivariable OR (95% CI)****	
Age							
<50 years old	157/92	102/41	0.76 (0.47, 1.23)	31/19	24/4	0.51 (0.10, 2.54)	0.43
≥50 years old	445/347	467/111	0.30 (0.22, 0.40)	69/69	106/14	0.12 (0.05, 0.26)	0.03
Sex							
Men	441/316	357/88	0.34 (0.25, 0.46)	78/65	82/9	0.14 (0.06, 0.37)	0.10
Women	161/123	212/64	0.48 (0.32, 0.74)	21/23	48/9	0.17 (0.05, 0.56)	0.11
BMI (kg/m^2)							
<25	404/295	386/105	0.38 (0.28, 0.52)	70/66	74/12	0.21 (0.09, 0.44)	0.10
≥25	198/144	183/47	0.36 (0.24, 0.55)	29/22	56/6	0.04 (0.01, 0.28)	0.08
Regular exercise							
No	291/290	173/107	0.64 (0.46, 0.88)	51/58	49/14	0.23 (0.09, 0.54)	0.03
Yes	308/149	393/45	0.20 (0.13, 0.30)	48/30	80/4	0.10 (0.03, 0.35)	0.17
Smoking status							
Never	251/195	270/71	0.35 (0.24, 0.51)	34/35	58/13	0.25 (0.10, 0.64)	0.37
Ever	351/244	299/81	0.41 (0.29, 0.58)	65/53	72/5	0.07 (0.02, 0.25)	0.006
Alcohol consumption							
Never	163/118	183/51	0.52 (0.32, 0.84)	28/31	45/11	0.23 (0.08, 0.63)	0.09
Ever	439/321	386/101	0.37 (0.28, 0.50)	71/57	85/7	0.08 (0.03, 0.24)	0.02

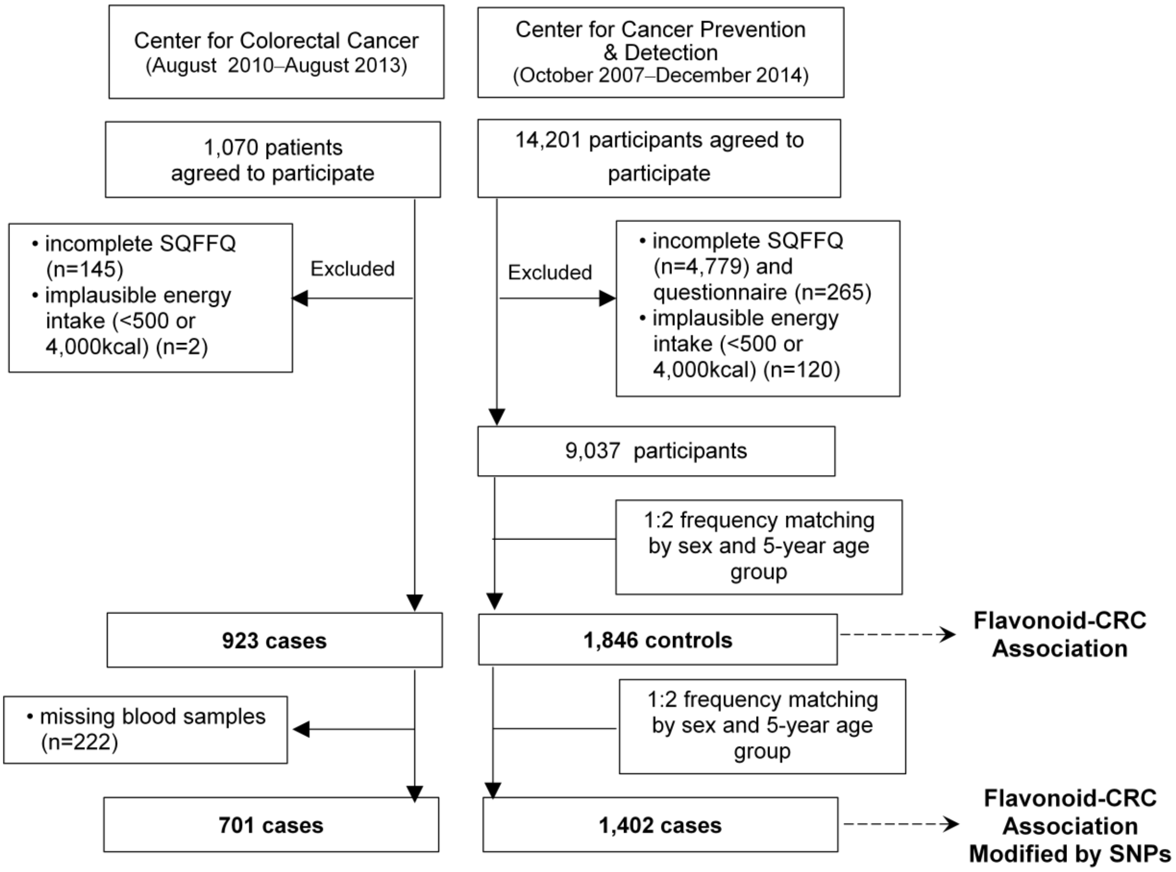

Supplementary Figure S1. Flow diagram of the selection of cases and controls
